# Strongly Fluorescent Blue-Emitting La_2_O_3_: Bi^3+^ Phosphor for Latent Fingerprint Detection

**DOI:** 10.3390/ma17174217

**Published:** 2024-08-26

**Authors:** Hanen Douiri, Marwa Abid, Lamia Rzouga Haddada, Layla Brini, Alessandra Toncelli, Najoua Essoukri Ben Amara, Ramzi Maalej

**Affiliations:** 1 Laboratory of Dielectric and Photonic Materials, Faculty of Sciences of Sfax, Sfax University, Sfax 3018, Tunisia; hanen.douiri.etud@fss.usf.tn (H.D.); marwa.abid.etud@fss.usf.tn (M.A.); layla.brini.etud@fss.usf.tn (L.B.); ramzi.maalej@fss.usf.tn (R.M.); 2 LATIS—Laboratory of Advanced Technology and Intelligent Systems, Higher Institute of Applied Sciences and Technology of Sousse, University of Sousse, Sousse 4011, Tunisia; lamiarzouga@yahoo.fr; 3 Dipartimento di Fisica, Università di Pisa, Largo B. Pontecorvo 3, I-56127 Pisa, Italy; 4 Istituto Nanoscienze CNR, Piazza San Silvestro 12, I-56127 Pisa, Italy; 5 Istituto Nazionale di Fisica Nucleare-Sezione di Pisa, Largo B. Pontecorvo 3, I-56127 Pisa, Italy; 6 LATIS—Laboratory of Advanced Technology and Intelligent Systems, National School of Engineers of Sousse, University of Sousse, Sousse 4023, Tunisia; najoua.benamara@eniso.rnu.tn

**Keywords:** phosphors, sol–gel combustion method, latent fingerprints, minutiae extraction

## Abstract

Blue-emitting bismuth-doped lanthanum oxide (La_2_O_3_: Bi^3+^) with various concentrations of Bi was synthesized using the sol–gel combustion method and used for visualization of latent fingerprints (LFPs). An X-ray diffraction (XRD) study revealed the hexagonal structure of the phosphors and total incorporation of the bismuth in the La_2_O_3_ matrix. Field Emission Scanning Electron Microscopy (FE-SEM) and Fourier Transform Infrared Spectroscopy (FTIR) were used to study the morphology and the relative vibrations of the synthesized samples. Photoluminescence (PL) studies showed strong blue emission around 460 nm due to the ^3^P_1_ → ^1^S_0_ transition. Clear bright-blue fingerprint images were obtained with the powder dusting method on various surfaces like aluminum, compact discs, glass, wood and marble. A first evaluation of these images indicated a clear visualization of all three levels of details and a very high contrast ranging from 0.41 on marble to 0.90 on aluminum. As a further step, we used an algorithm for extracting fingerprint minutiae with which we succeeded in detecting all three levels of fingerprint details and even the most difficult ones, like open and closed pores. According to these analyses, La_2_O_3_: Bi phosphor is demonstrated to be an effective blue fluorescent powder for excellent visualization of latent fingerprints.

## 1. Introduction

The detection and comparison of fingerprints is the most used biometric technique for personal identification, as they are an invaluable source of information for forensic scientists [1,2,3]. However, at crime scenes, fingerprints are not apparent to the naked eye. Furthermore, fingerprints left on smooth surfaces are especially difficult to locate. Only level 1 and level 2 ridge features are typically seen, although a small number of individuals are able to see level 3 ridge details or sweat pores [4,5,6]. Hence, they are referred to as latent fingerprints (LFPs) and require particular processes to make them sufficiently apparent [7], such as fuming, powder dusting and single-metal deposition methods [8]. For these reasons, it is critical to boost the effectiveness of the powder dusting method, which is the most popular and straightforward approach for visualizing fingerprints as compared to alternative methods like silver nitrate spraying and ninhydrin fuming [6,9]. Iodine, cyanoacrylate and multi-metallic deposition are examples of physical/chemical approaches that have been applied to enhance fingerprint detection [10,11].

In the last several decades, fluorescent materials have shown good potential in forensic applications, particularly in latent fingerprint detection for the development of fingerprints, due to their unique optical properties [12,13,14,15]. Indeed, by being excited at particular wavelengths, they can produce intense fluorescence. Hence, some of the most successful applications of fluorescent nanomaterials are biolabeling and bioimaging [16,17]. These properties have led researchers to suggest using fluorescent particles to create latent fingerprints [18,19], guaranteeing minimal toxicity, high developing selectivity, high developing sensitivity and high developing contrast [20]. Moreover, in order to minimize interaction with different possible background colored surfaces, it is important to develop fluorescent agents with various color tones [21].

Rare earth (RE) elements are thought to be important as promising activators for fluorescent materials at various wavelengths due to their electronic structures, since their luminescence features rely on the location of the lanthanide (Ln) dopants’ 4f energy levels in relation to the valence band (VB) and conduction band (CB) of the host [22,23]. Among all rare earth metals, lanthanum oxide (La_2_O_3_) is a basic rare earth oxide that has been extensively used in research due to its novel optical, catalytic, electronic, magnetic and mechanical properties [24,25,26,27,28]. La_2_O_3_ has also been investigated as a phosphor host for lanthanide ions [29], such as Yb^3+^, Tb^3+^ [30] and Eu^3+^ [31].

Bismuth (Bi) ions exhibit interesting optical properties, yet they have been less intensively studied compared to rare earth ions. Bismuth has gained a tremendous amount of attention, since it is a non-toxic post-transition metal with a large number of possible valence states, which, together with its tendency to form clusters, make it a versatile but complex dopant for possible luminescence applications, such as LED ones [32].

In this work, we report the successful preparation of non-toxic powder based on highly luminescent blue-emitting La_2_O_3_:Bi^3+^ phosphors for fingerprint detection through a facile sol–gel combustion method without the need for the intervention of a fuel. It includes inorganic polymerization reactions like hydrolysis, polycondensation, gelation, aging, drying and calcination. Hence, this method has been widely used for the synthesis of different hybrid and doped materials, especially nanomaterials [33,34,35]. Crystal structure and luminescence properties were studied as a function of the concentration of Bi^3+^ ions (0.2, 0.5 and 1 mol %). Furthermore, we applied the optimized La_2_O_3_:Bi^3+^ phosphors to identify fingerprints on a variety of surfaces. We selected a number of frequently used items from our everyday collection, including CDs, aluminum foil, wood, ceramics and glass, on which fingerprints are frequently left. The quality of the retrieved minutiae was finally analyzed using a fingerprint minutiae detection method. The analyses verified that, once a decent identification of their minutiae is established, the co-doped phosphors can be useful for the imaging of latent fingerprints.

## 2. Materials and Methods

### 2.1. Material Synthesis and Characterization

Powder samples of La_2_O_3_:Bi^3+^ with different concentrations were prepared by the citric acid sol–gel combustion synthesis technique. The starting materials (La (NO_3_)_3_·6H_2_O (99.999%), citric acid monohydrate (C_6_H_8_O_7_·H_2_O, 99.5%, analytical grade) and Bi (NO_3_)_3_·5H_2_O (99.999%)) were purchased from Sigma Aldrich (St. Louis, MO, USA). A quantity of 50 mL of distilled water was used to thoroughly dissolve 5 g of citric acid monohydrate and 5 g of lanthanum nitrate. After that, an appropriate amount of bismuth nitrate (Bi (NO_3_)_3_·5H_2_O) was added to the previously stated solution dropwise after being separately dissolved in 10 mL of distilled water and 0.5 mL of 70 % concentrated nitric acid. After three hours of intense stirring at 85 °C, this was placed in a 200 °C furnace and burned with a white flame for 2 h. Brown powder was the end outcome. In order to improve the crystallinity and to reduce the impurities of the synthesized phosphor, it was heat-treated in an oven at a temperature of 1200 °C, maintained for 2 h.

A phase composition analysis of the prepared compounds was performed using an X’Pert PRO MPD diffractometer (Malvern Panalytical, Malvern, UK) with a Cu Kα (λ = 1.5419Å). Scanning was in the 2θ range of 20–80°. Surface morphologies of all samples were examined using a field emission scanning electron microscope (TESCON-Mira III XMU) (Kohoutovice, Czech Republic) equipped with an energy dispersive spectrophotometer (EDS). The Fourier transform infrared (FT-IR) spectra of the compounds were recorded at room temperature on a Perkin Elmer Spectrum (Waltham, MA, USA) in the range of 500–4000 cm^−1^. The room-temperature photoluminescence (PL) spectra were recorded using a Shimadzu Spectrofluorophotometer (RF-6000) with a 150 W Xenon lamp (Duisburg, Germany). The CIE chromaticity coordinates were calculated using the emission spectra.

### 2.2. Latent Fingerprint Feature Extraction

Fingerprints hold significant importance in forensic science and are extensively studied due to their distinctive patterns and enduring characteristics [36,37,38]. The features of fingerprints are categorized into three levels:

- Level 1: This level consists of coarse features like arches, loops and whorls.
- Level 2: This level involves identifying irregularities, known as minutiae, on capillary lines. These minutiae are then categorized into four types: terminations, bifurcations, islands and lakes.
- Level 3: This level involves microscopic details, including pores.

**Figure 1:**
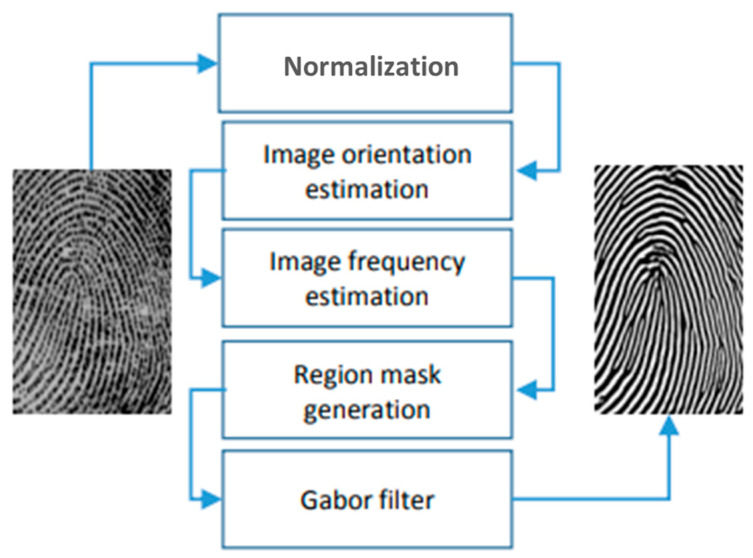
Description of the preprocessing phase.

These intricate fingerprint characteristics serve the purpose of identifying individual latent fingerprints, which are the impressions created when the grease and sweat on a finger transfer from the ridges to a contact surface upon touching it. Extensive research is conducted on fingerprints, owing to their distinctive patterns and enduring characteristics [37]. The visualization of latent fingerprints plays a crucial role in identifying both crime scenes and personal information. Thus, in order to demonstrate the satisfactory performance of the prepared La_2_O_3_: Bi phosphor as a fingerprint labeling agent, we chose to employ a fingerprint minutiae extraction algorithm to assess both the visualization and the quantity of fingerprint images. The algorithm in question, detailed in [36], involves preprocessing and minutiae extraction, as outlined below:

- Preprocessing: This involves enhancing the image through a series of five main steps aimed at improving image quality for more effective feature extraction (Figure 1). The process begins with image normalization, followed by orienting the peaks of the fingerprint and estimating the frequency. Subsequently, a segmentation step is applied to identify the peak regions in a fingerprint image, creating a mask to delineate the region and normalizing image intensity values. Ultimately, oriented Gabor filters are utilized.
- Feature extraction: The process of extracting minutiae features commences with an enhanced grayscale image. Following image binarization, a morphological thinning technique is applied to reduce ridge structures to a single pixel width, creating what is referred to as the skeleton. Subsequently, every pixel of the thinned binary image is analyzed to identify minutiae locations.

## 3. Results

### 3.1. Structure and Morphology: XRD, SEM and EDX Studies

**Figure 2:**
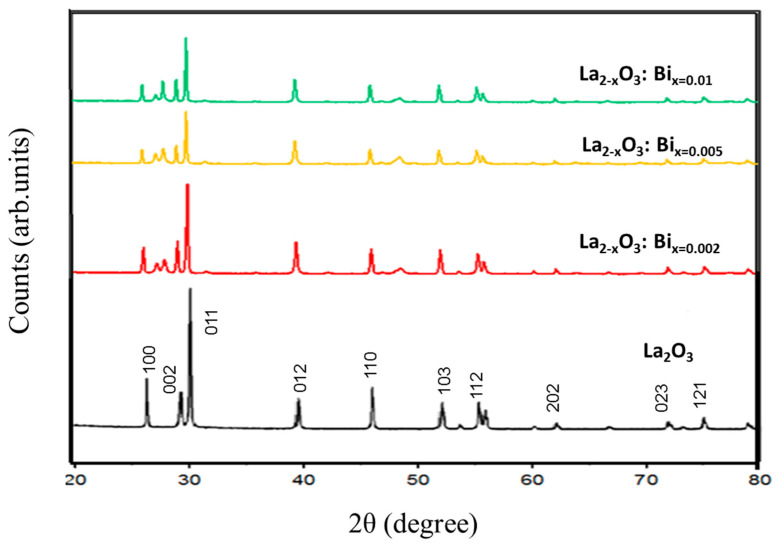
La_2_O_3_ and La_2-x_O_3_: Bi_x_ powders (x = 0.002, 0.005 and 0.01).

The XRD patterns of La_2-x_O_3_: Bi_x_ powder samples with x = 0.002, 0.005 and 0.01 are shown in Figure 2. On the basis of the Joint Committee on Powder Diffraction Standard (JCPDS) reference database, the diffraction peaks of the precursor are well indexed to hexagonal-phased La_2_O_3_ (JCPDS no. 05-0602). For synthesized samples, there is no variation in the location of diffraction peaks at different doping levels. However, additional diffraction angles of 27°, 27.7° and 48.5° located at 2θ = 27, 48 and 50 correlate with the (110), (101) and (211) and planes related to the presence of an La (OH)_3_ phase [39].

**Figure 3:**
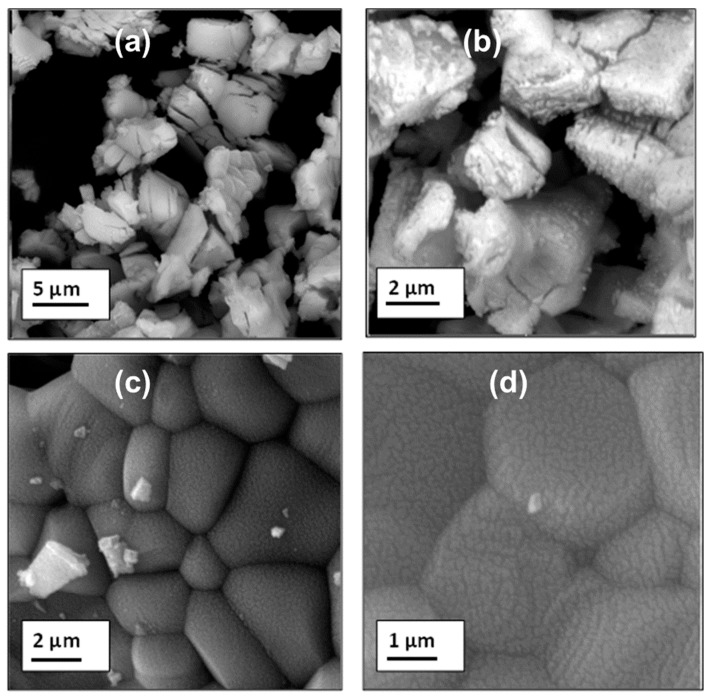
SEM micrographs of prepared La_2_O_3_ Bi phosphors: (**a**,**b**) for x = 0.002; (**c**,**d**) for x = 0.01.

Figure 3 shows SEM images of the prepared La_2_O_3_: Bi^3+^ phosphors (x = 0.002 and 0.01). The particles of La_2_O_3_ have an irregular shape, and each particle contains many crystallites. However, some minor agglomerations can be observed, which is due to the fact that the Bi^3+^ ions influence the growi interface and change the chemical bonding.

**Figure 4:**
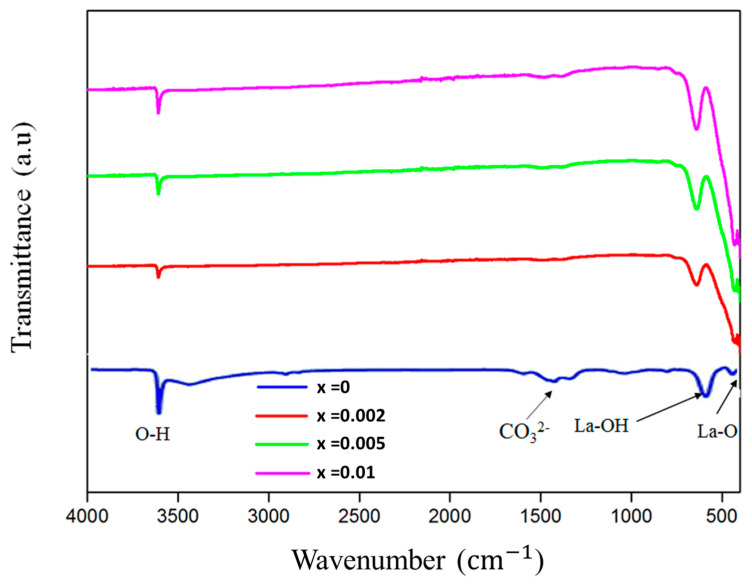
FTIR spectra of the La_2_O_3_ and La_2-x_O_3_: Bi_x_ powders.

The FTIR spectra in Figure 4 correspond to the samples prepared with different concentrations of bismuth (x = 0, 0.02, 0.05 and 0.1). The samples showed similar FTIR spectra with increasing peak intensities. Strong absorption and abrupt peaks at 3610 ${cm}^{-1}$ could be attributed to stretching O–H vibrations brought on by moisture absorbed from the environment [40]. The appearance of several peaks in the range of $1000–1625\;{cm}^{-1}$ may be attributed to the ν_3_ and ν_1_, ν_2_, and ν_4_ modes of the ${CO}_{3}^{2-},$ which confirms the presence of CO_2_ absorbed from the atmosphere [41]. The absorption peaks at about 645 ${cm}^{-1}$ may be attributed to La–OH vibration, and the small band at around 430 ${cm}^{-1}$ may be assigned to the characteristic metal oxide La–OH vibration [42].

### 3.2. Photoluminescence (PL) Properties

**Figure 5:**
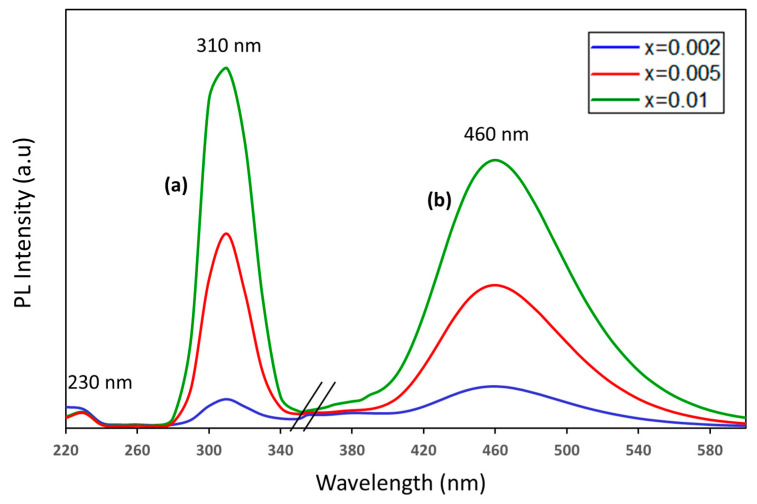
(a) Excitation and (b) emission spectra of La_2-x_O_3_: Bi_x_ doped with different Bi concentrations.

Figure 5 shows the PL excitation and emission spectra of the La_2_O_3_ doped with different Bi concentrations. The excitation spectra of the blue emission (around 460 nm) consisted of two peaks at 230 nm and 310 nm corresponding to transitions from the ^1^S_0_ ground state to the ^1^P_1_ and ^3^P_1_ energy levels, respectively. On the other hand, broadband blue emission was observed at around 460 nm after 310 nm excitation. These Bi^3+^ ions’ radiative characteristics are attributed to parity-allowed transitions between the 6s^2^ ground state (^1^S_0_) and the 6s6p excited states, which are represented by the states ^3^P_J_ (J = 0–2) and ^1^P_1_. While the optical transition from ^1^S_0_ to ^1^P_1_ is spin-allowed, the optical transitions from ^1^S_0_ to ^3^P_0_ and ^3^P_2_ are totally spin-forbidden. The ^3^P_1_ level is permitted when the ^1^P_1_ and ^3^P_1_ levels are mixed via spin–orbit coupling [43].

Additionally, in Bi^3+^-doped materials, a lower energy luminescence is sometimes reported in addition to the characteristic broad band ^3^P_0,1_ → ^1^S_0_ luminescence. It might result from a charge transfer transition between metals (CB → Bi^3+^/Bi^4+^) as well as from Bi^3+^-Bi^3+^ pairs or clusters [44,45,46].

For the emission spectra, a single broad band of blue emission centered at 460 nm was detected and attributed to radiative transitions from the ^3^P_1_ excited state back to the ^1^S_0_ ground state. Moreover, the photoluminescence intensity increased with increasing Bi^3+^ ion concentration.

### 3.3. CIE Chromaticity

**Figure 6:**
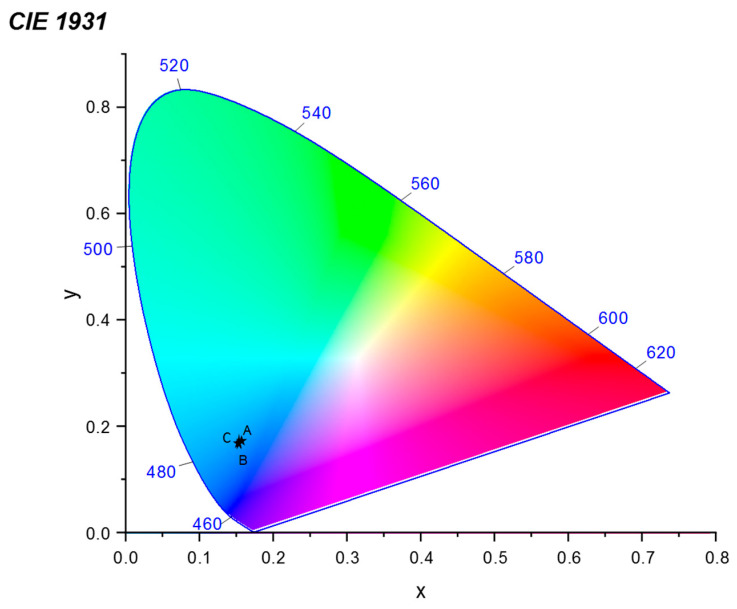
CIE color chromaticity coordinates of La_2_O_3_: Bi^3+^ phosphors: A (x = 0.2), B (x = 0.5) and C (x = 1).

Color coordinates of the phosphors were calculated using the Commission Internationale de l’Eclairage (CIE) 1931 color chromaticity diagram. Figure 6 represents the CIE diagram of La_2_O_3_: x% Bi (x = 0.2, 0.5 and 1) phosphors. The CIE color coordinates of all the samples are almost equal and lie around (0.157, 0.172), which is very close to that of the ideal blue light.

### 3.4. Latent Fingerprint Detection Using the Powder Dusting Method

**Figure 7:**
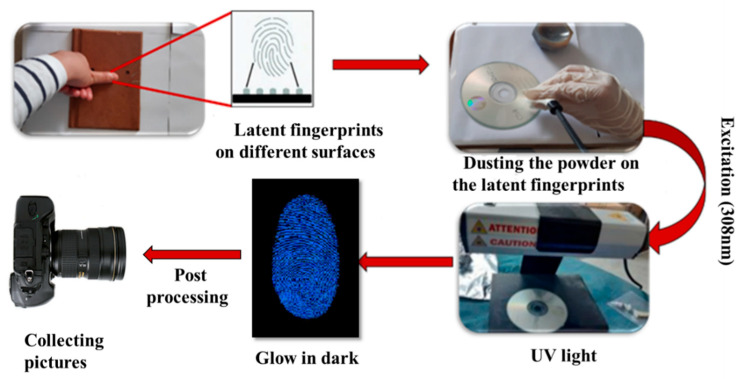
Illustration of the development of latent fingermarks using the 1%Bi: La_2_O_3_ phosphor dusting process.

Good visualization of the latent fingerprints (LFPs) on various surfaces was anticipated based on the photoluminescence results. Thus, to test our novel powders, both porous and non-porous surfaces (such as glass, plastic, aluminum foil and compact discs) were chosen. The donor’s hand was thoroughly cleaned and dried before the imprint was made. The donor’s fingerprints were then carefully wiped off their foreheads and their clean fingertips were pushed against a variety of room-temperature objects. After that, the LFPs were dyed with Bi-doped La_2_O_3_ using a soft feature brush and a smooth brushing technique. Finally, new fingerprints were developed and prepared for visualization. The production of fluorescent powder, which enables latent fingerprints to be seen under UV light, was the primary objective of this experiment. Figure 7 displays a schematic view of the development of the fingerprints.

**Figure 8:**
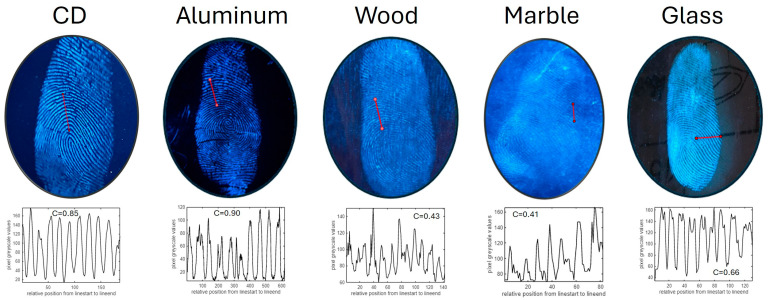
Photographs (under 302 nm UV-illumination) of LFPs on different substrates developed by La_2_O_3_: Bi^3+^ fluorescent blue powder. For every picture, the line intensity profile along the red line and the best contrast obtained are shown at the bottom.

Figure 8 shows the images of fresh fingerprints under UV lamp illumination (λ = 302 nm) developed by La_2_O_3_: 1% Bi^3+^ phosphors on CD, aluminum foil, wood, marble and glass surfaces, respectively. In general, the quality of the LFPs developed on different surfaces, based on visual assessment, was excellent, and information of levels 1–3 could be easily seen. In all cases, we extracted the intensity profile along a line after conversion of the RGB picture to grayscale. The intensity profile was then used to calculate the intensity fringe contrast as follows:

$$C=\frac{I_{MAX}-I_{min}}{I_{MAX}+I_{min}}$$

where $I_{MAX}$ and $I_{min}$ are the maximum and minimum intensities in the line profile, respectively. As expected, the lowest contrasts were obtained on difficult and porous surfaces, namely, wood and marble (0.43 and 0.41, respectively); the contrast on glass was 0.66; on CDs, it was 0.85; and the highest contrast of 0.90 was obtained on aluminum.

**Figure 9:**
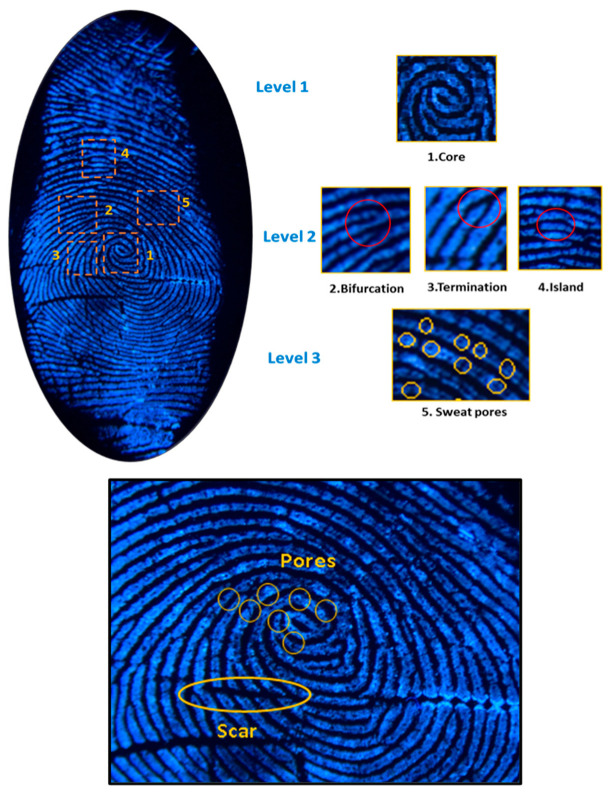
Latent fingerprint image visualization (under 302 nm UV illumination) displaying first-, second- and third-level details.

Figure 9 shows an enlarged fingerprint picture developed on aluminum foil under UV light (λ = 302 nm). All three levels of fingerprint ridge patterns are clearly visualized: the core (level 1); bifurcations, terminations and islands (level 2); and sweat pores (level 3).

Actually, general morphological details like orientation fields, ridge patterns and fingerprint ridge flows are level 1 details. Information regarding the pattern agreement of each fingerprint’s ridges is provided by level 2 details. Sweat pores, curvature and dots are examples of the dimensional fingerprint ridge features that are classified as level 3 details [47].

### 3.5. Automatic Minutiae Extraction

**Figure 10:**
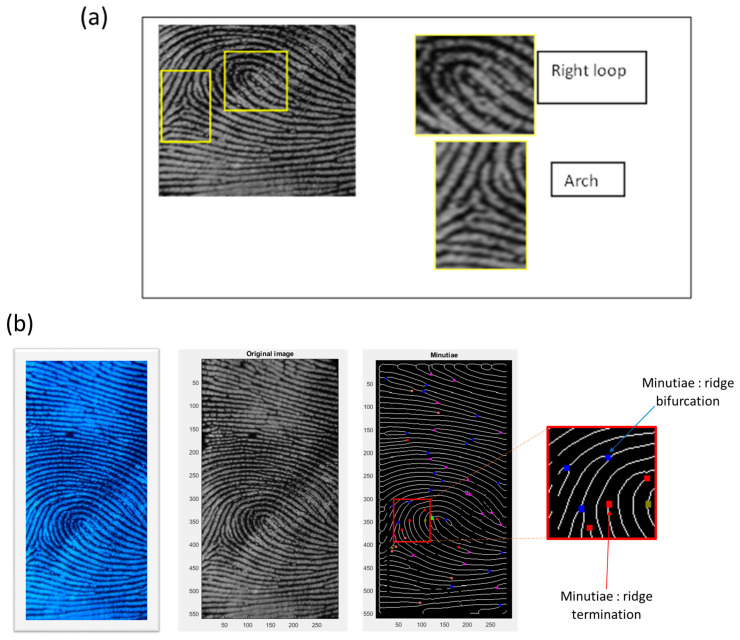

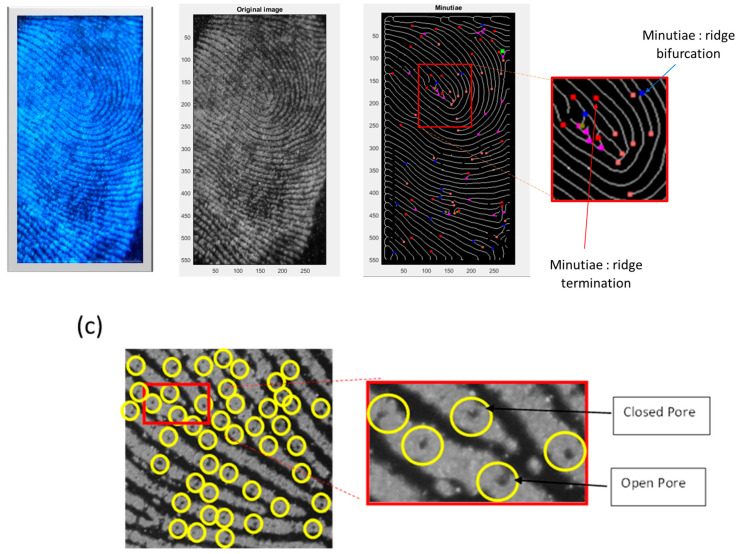
The latent fingerprint image features evidenced by the 1% Bi, La_2_O_3_ phosphors on aluminum foil under UV light based on an automatic algorithm ((**a**) level 1, (**b**) level 2, and (**c**) level 3).

Macroscopic and microscopic details play a crucial role in personal identification. In order to demonstrate the feasibility of the La_2_O_3_: Bi phosphor as a fingerprint developing agent, we opted for the fingerprint minutiae extraction algorithm developed in [36] and performed assessments using the BioSecure, FVC DB1 and PolyU public databases [37]. The detected latent fingerprint images stained with La_2_O_3_: Bi on compact disc, aluminum, wood, marble and glass surfaces could be well visualized, and details of levels 1–3 were well identified. Figure 10 shows some examples of automatic identification of loops, arches, ridge terminations, and open and closed pores.

## 4. Conclusions

In summary, a facile sol–gel combustion method was employed to conveniently synthesize fluorescent La_2_O_3_: Bi^3+^ (0.2, 0.5 and 1 mol %) phosphors for latent fingerprint detection. According to a structural and spectroscopic analysis, the acquired samples had a good crystalline structure, indicating that the Bi^3+^ ions were effectively incorporated into the crystalline hexagonal structure of the La_2_O_3_ matrix. A photoluminescence study showed a strong broad band of blue fluorescence emission centered at 460 nm for excitation at 310 nm, corresponding to the radiative transition of the ^3^P_1_ excited state back to the ^1^S_0_ ground state. The CIE color coordinates were evaluated, and it was found that the photoluminescence emission from La_2_O_3_: Bi^3+^ phosphors lay in the blue region. By employing the powder dusting technique, when exposed to UV light, the blue emission from La_2_O_3_: Bi phosphors demonstrated significant promise for the viewing of latent fingerprints by generating latent fingerprint images with excellent contrast and recognition of all three levels of features on a variety of materials, including challenging ones like wood and marble. In parallel, an evaluation based on an automatic algorithm was performed by extracting fingerprint minutiae. We succeeded in detecting level 3 details corresponding to open and closed pores. The results of this work demonstrate the great potential of La_2_O_3_: Bi^3+^ phosphors for latent fingerprint feature extraction either in terms of visual evaluation or evaluation based on automatic recognition, even with a low Bi doping.

## Data Availability

The raw data supporting the conclusions of this article will be made available by the authors on request.
